# Multiscale Analysis of the Highly Stretchable Carbon−Based Polymer Strain Sensor

**DOI:** 10.3390/polym15071780

**Published:** 2023-04-03

**Authors:** Junpu Wang, Zhu Wang, Yanjiang Zuo, Wenzhi Wang

**Affiliations:** 1 College of Mechanical and Electrical Engineering, Shaanxi University of Science and Technology, Xi’an 710021, China; wangjunpu@sust.edu.cn (J.W.);; 2 School of Aeronautics, Northwestern Polytechnical University, Xi’an 710072, China

**Keywords:** keyword flexible strain sensor, carbon nanoparticle−filled polymer, representative volume element, multiscale analysis

## Abstract

In this paper, a multiscale analysis method was proposed to simulate carbon nanoparticles (CNPs)−filled polymers which can be strain sensors applied in wearable electronic devices, flexible skin, and health monitoring fields. On the basis of the microstructure characteristics of the composite, a microscale representative volume element model of the CNPs−filled polymer was established using the improved nearest−neighbor algorithm. By finite element analysis, the variation of the junction widths of adjacent aggregates can be extracted from the simulation results. Then, according to the conductive mechanism of CNP−filled polymers, the composite was simplified as a circuit network composed of vast random resistors which were determined by the junction widths between adjacent aggregates. Hence, by taking junction widths as the link, the resistance variation of the CNPs−filled polymer with the strain can be obtained. To verify the proposed method, the electromechanical responses of silicone elastomer filled with different CNPs under different filling amounts were investigated numerically and experimentally, respectively, and the results were in good agreement. Therefore, the multiscale analysis method can not only reveal the strain−sensing mechanism of the composite from the microscale, but also effectively predict the electromechanical behavior of the CNPs−filled polymer with different material parameters.

## 1. Introduction

In recent years, the demand for flexible and stretchable strain sensors has been rapidly growing due to their potential applications in wearable electronics, health monitoring, and soft robots [1,2,3,4,5]. An effective method is to add a conductive filler to the insulting−material matrix to endow the material with sensing property. As one of them, carbon−filled polymers have attracted a lot of attention. Yellapantula et al. [6] developed a flexible sensor array made of a carbon nanoparticles (CNPs)−filled rubber composite to determine the object placed on the top of the sensor by capturing the change in instantaneous pressure. Similarly, highly elastic and flexible multilayered carbon black/elastomer composite−based capacitive pressure/force sensor arrays were fabricated for soft robotics [7]. The sensor had a wide range of applications owing to the high sensitivity of 150 pf/MPa, fast response times, and low sensor drift under different loading conditions. In addition, the carbon−based polymer showed great potential as a robotic tactile sensor for monitoring human behavior. Lv et al. [8] presented a facile and green surface modification strategy of carbon nanotubes (CNTs) to fabricate natural rubber/CNTs composites, which can potentially be applied to accurately monitor both large and small strain behaviors in human motions. In addition, the strain sensor prepared using solution mixing graphene, CNPs and silicone rubber was shown to have high strain and sensitivity, so it can effectively detect pulse beating, swallowing, muscle contractions, and other movements [9]. Moreover, a multi−walled CNTs sensor spread in silicone rubber has been proposed to measure the water flow rate [10], as the electrical resistance of the elastic sensor varies with changes in fluid pressure in pipes.

Despite the fact that carbon−based polymer sensors have received widespread attention, current research has focused mainly on the manufacture of the device and the sensing behavior [11,12,13,14] while research on its sensing mechanism is limited. In the initial research, an analogy method was proposed to quantitatively describe the electromechanical behavior of silicone rubber filled with CNPs, but the sensing mechanism was not involved [15]. Furthermore, based on the conductive mechanism of carbon−based polymers, the electrical resistance and strain have been connected by simplifying the microcircuit network and tunneling theory [16,17]. However, because the fillers are random distributions in polymer, researchers have described the relationship between the deformation and microstructural parameters roughly by some hypotheses or random functions. With the development of finite element (FE) analysis, this has also been used in the electromechanical investigation of carbon−based polymers [18,19,20,21] An FE approach has been presented to model the conductivity and strain−sensing capabilities of a CNT−reinforced polymer composite with a periodic representative volume element (RVE). The model was established based on the measurable statistical descriptors of the microstructure [22]. In addition, Yang et al. constructed an RVE with randomly distributed graphene in a rubber matrix to predict the piezoresistive properties and calculate the strain−sensing behavior of graphene rubber composites [23]. 

Similarly, to describe the electromechanical coupling behavior of carbon−based polymers under large deformation, the authors have attempted to establish an analytical and numerical method to simulate the CNPs−filled polymer by the FE method in previous work [24], but part of the microstructural parameters was ignored because the model was simplified to two dimensions (2D). Therefore, in this paper, the approach is improved by modifying the modeling method to reveal the strain sensing mechanism and predict the electromechanical behavior of the carbon−based polymers with different material parameters. Taking CNPs−filled polymer as an example, the resistance variation of the composite with the deformation was obtained through the link of junction widths that could be extracted by finite element analysis of a 3D RVE model. Compared to the previous approach, the developed model contains more details, so it is closer to the real microstructure of the composite. Using this method, we predicted the electromechanical behavior of CNPs−filled polymers under different parameters in this paper. Meanwhile, the resistance–strain curves of the conductive polymer were obtained with the uniaxial tensile test. Finally, the multiscale analysis method presented in this paper is validated by comparing the simulation results with the experimental data.

## 2. Methods

**Figure 1:**
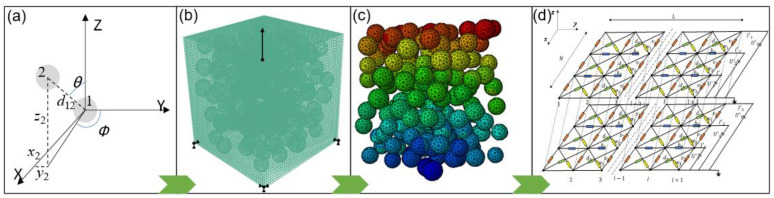
Steps of the multiscale analysis method: (**a**) improved NNA method; (**b**) RVE model; (**c**) FE analysis; (**d**) equivalent resistance calculation.

On the basis of the conductive mechanism of the CNPs−filled polymer, the composite can be simplified as a circuit network comprised of vast random resistors, which were determined by the junction widths between two nearby CNP aggregates. To describe the random distribution of the conductive aggregates, a 3D RVE model was established using the improved NNA method. By FE analysis, the variation of the distance between adjacent aggregates with different elongations could be obtained. Then, based on tunneling theory, the equivalent resistance variation of the conductive composite could be calculated by the admittance matrix method. The steps of the multiscale analysis method are shown in Figure 1.

### 2.1. Establishment of the RVE Model

Thanks to the random distribution of CNPs in the polymer matrix, the internal structure of the composite presents a non−uniform feature. To describe the microstructure of the composite, the RVE model was generated, which is a typical means for the mechanical analysis of carbon−based elastomer on a microscale [25,26]. The 2D RVE random model is usually adopted for composite FE analysis, and the conductive particles or aggregates are limited to a plane to form a conductive path. The information presented from the 2D model is not enough to reflect the macro−performance of the materials. In order to be closer to the real structure of the composite, in this paper a 3D RVE model of CNPs−filled polymer was established. Compared to the 2D model established in our previous work, it contained more structural details and higher randomness.

**Figure 2:**
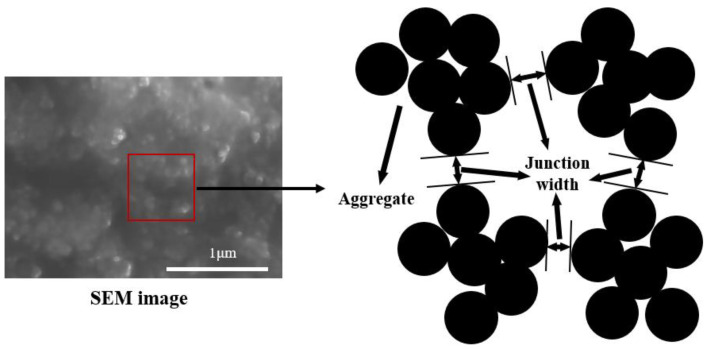
Schematic diagram of CNPs−filled polymer. (The red box means the local area of the SEM image can be represented by the schematic diagram on the right.)

The CNPs usually formed aggregates in polymer, and the particles were gathered into an irregular sphere, as shown in Figure 2. However, to simplify the parameters and facilitate modeling, the aggregate was approximately abstracted as a sphere in the RVE model, which is generated based on some assumptions.

Firstly, the aggregates are assumed to be uniformly distributed in the matrix, and the spatial coordinates and radii of the aggregates are randomly generated. To indicate the size differences, the spherical diameters of the aggregates have a random variation of ±5%. In addition, the spheres cannot overlap or intersect; that is, the aggregates are considered as hard−core structures.

**Figure 3:**
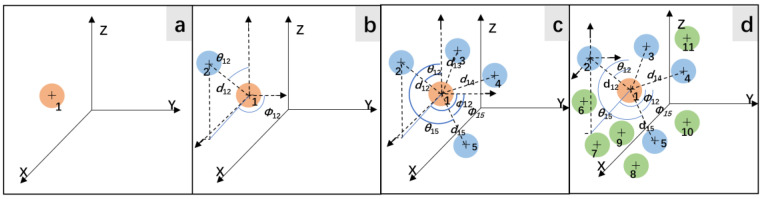
Schematic diagram of the improved NNA algorithm. (**a**–**d**) show the generation flow diagrams of the improved NNA algorithm.

To represent the realistic random microstructure of the composite, the common approach is to generate statistically equivalent RVE, such as a random sequential adsorption model (RSA) [27] and nearest−neighbor algorithm (NNA) [28]. Additionally, a new algorithm is proposed based on NNA to bring the RVE model more in line with microstructure characteristics and also make it apply to the composite of the higher volume fraction [29]. In this paper, a 3D coordinate method is proposed to generate CNPs aggregates based on the improved NNA. The generation process is shown in Figure 3, where ($x_{i}$,$y_{i}$,$z_{i}$) is the spatial coordinate of the *i*th aggregate and *φ*, *θ*, *d* are the azimuth, elevation, and distance, respectively. The aggregates generated in different steps are distinguished by different colors.

a. Randomly generate the 3D coordinates ($x_{1}$,$y_{1}$,$z_{1}$) and radius *r*_1_ of the first aggregate.
b. Randomly generate the distance *d*_12_, azimuth *φ*, and elevation *θ*, and the coordinates of the second aggregate ($x_{2}$,$y_{2}$,$z_{2}$) can be derived by
  (1) $$\left\{\begin{array}{l} x_{2}=x_{1}+d_{12}cos\theta sin\varphi \\ y_{2}=y_{1}+d_{12}cos\theta cos\varphi \\ z_{2}=z_{1}+d_{12}sin\theta \end{array}\right.$$

Then, check the space distance between the first and second aggregates. If it is smaller than the given minimum value, delete the newly generated aggregate and regenerate.

c. Generate more aggregates in the vicinity of the first aggregate.
d. Repeat the previous steps until the desired volume fraction is reached.

### 2.2. Conductive Mechanism of CNPs−Filled Polymer

**Figure 4:**
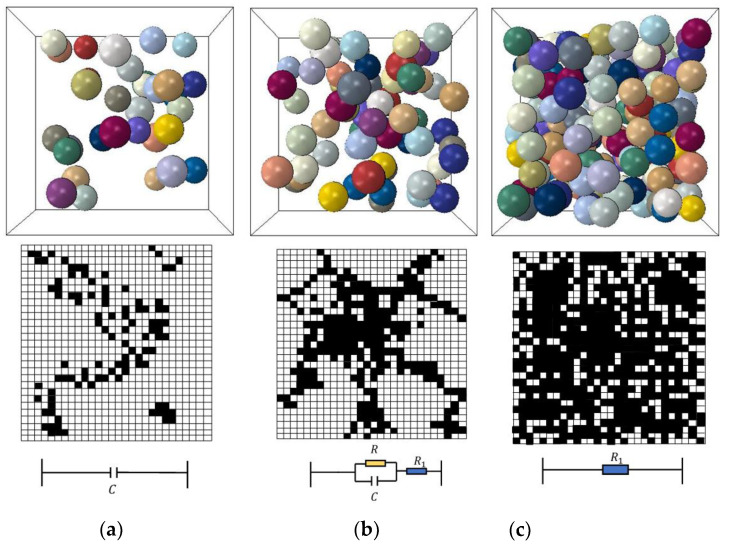
Relationship between the filler amount and the conductivity (*R* and *C* represent the resistance and capacitance between two adjacent aggregates, respectively, and *R*_1_ is the resistance across the aggregates): (**a**) Insulation region; (**b**) Percolation region; (**c**) Conductive region.

The conductive mechanism of CNPs−filled composites is complex. According to percolation theory, the conductivity of the composite is related to the conductive path formed by the filled aggregates. Hence, the resistivity of CNPs−filled composite is related to the amount of filling. With the increase of the conductive filler, the resistance of the composite changes through three stages (as shown in Figure 4). When the amount of filling was small, the probability that the conductive aggregates will contact each other was very small. The effective conductive path in the composite was discontinuous, and the composite was considered a network composed of many capacitors, so the composite shows an insulation state. With the increase of conductive fillers, the junction width between aggregates gradually reduces and the conductivity of the composite improves. When the filling amount reaches the threshold, the conductive path will be connected, and the composite will be conductive. The conductive path can be regarded as a network composed of the capacitance, the resistance of the conductive CNP aggregates, and the tunneling resistance, which was determined by the junction width of the adjacent conductive aggregates. When the filling amount is greater than the threshold, the resistivity of the composite continues to decrease because of the reduction of the junction widths between the aggregates. If the filling amount is much greater than the threshold, the resistivity changes little because the variation of the junction width is limited. 

**Figure 5:**
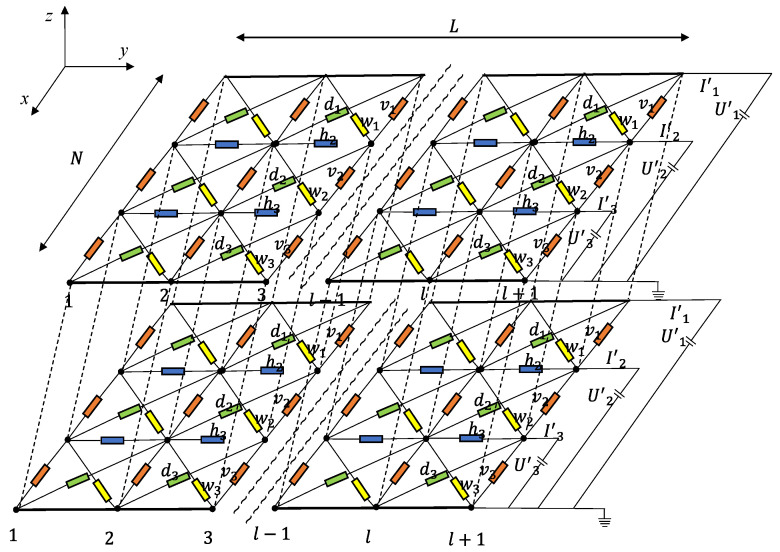
Three−dimensional infinite circuit network.

In this paper, due to the composite employed under DC voltage, the capacitance was equivalent to an open circuit, and the resistance of the composite was represented as R+R1. Moreover, the resistance in the R1 aggregates was neglected because the conductivity of the CNPs was much greater than the polymer matrix. Therefore, the CNPs−filled polymer can be regarded as an infinite circuit network formed by multiple tunneling resistors in series and parallel. Xie et al. [17] simplified the conductive composite as a 3D infinite circuit network with many random resistors (as shown in Figure 5). The composite was assumed to have multiple 2D film layers in parallel connection along the thickness direction, and there were no differences between each layer. N and L are the numbers of vertical and horizontal resistors, respectively, and $h_{i}$,$v_{i}$,$d_{i}$, and $w_{i}$ denote the unit cell resistors.

The circuit network consists of numerous random resistors that obeyed the tunneling conduction theory. In the low voltage region, the tunneling resistance R between two adjacent aggregates can be approximately expressed as [30]:

(2) $$R=\frac{2h^{2}\Delta }{3e^{2}S\sqrt{2m\varphi }}*exp\left(\frac{4\pi \Delta }{h}\sqrt{2m\varphi }\right)$$

where *h* is Planck’s constant, *S* represents the cross−sectional area of the aggregates, and *e*, *m*, *φ*, and Δ denote the electronic charge, the mass of the electron, the height of the potential barrier and the width of the junction, respectively. Therefore, the junction width is the key parameter for calculating the resistance of the composite.

## 3. Simulation

According to the proposed method, we predicted the electromechanical properties of the CNPs−filled elastomer under uniaxial tensile loading with different parameters, such as different fillers and different filling amounts. Silicone rubber was selected as the matrix because it is flexible, stretchable, and easily obtained by room−temperature vulcanization. The fillers were Ketjenblack EC−600JD and EC−300J, two commonly used superconductive CNPs. The parameters of CNPs and silicone rubber are shown in Table 1 and Table 2, respectively.

In CNPs−filled silicone elastomers, the particles usually exist as aggregates, and the average diameter of the aggregates $d_{m}$ is considered to be related to the specific surface area *S* and density $\rho_{c}$ in industry.

(3) $$d_{m}=\frac{K}{\rho_{c}S}$$

where *K* is the shape factor, and for spherical or cubic models, it usually equals 6.

For the compound polymer, the relationship between the volume fraction of the filler *φ* and the weight can be expressed as

(4) $$\varphi =\frac{\psi /\rho_{c}}{100/\rho_{rubber}+\psi /\rho_{c}}$$

where *ψ* is the weight part of the conductive filler added to 100 phr of rubber.

**Figure 6:**
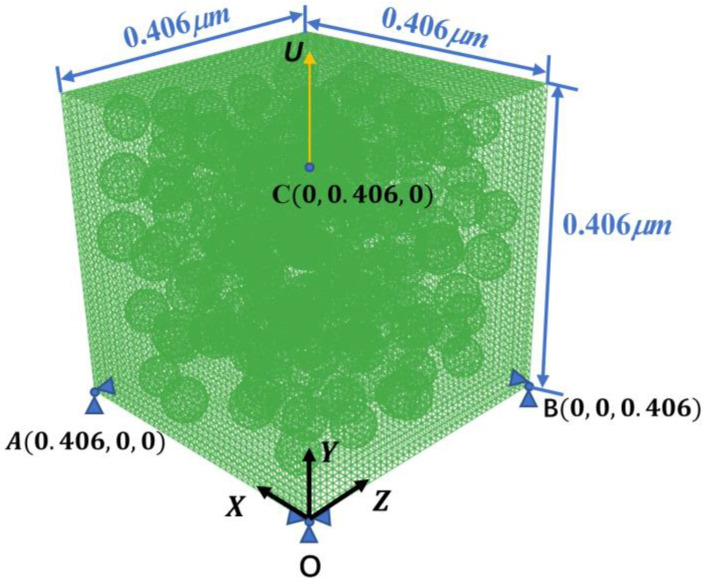
The 3D RVE model of silicone elastomer filled with EC−300J CNPs with 30% volume fraction.

According to the random distribution data obtained from the improved NNA, the RVE model of the CNPs−filled silicone composite was established by the commercial software ABAQUS. As is known, the model with a larger size contains more microstructure information, but excessive size will lead to an excessive number of meshes, which will increase the calculation cost. Therefore, the appropriate model size can improve the efficiency of the calculation based on ensuring the accuracy of the results. The feature size of the model, which can be expressed as the ratio of the edge length and the radius of the aggregates, has been shown to be more consistent with the experimental data when it is equal to or greater than 14 [31]. Hence, considering the calculation time, the feature size was taken as 14 in this paper and the edge length of the model was calculated. Taking the silicon elastomer filled with EC−300J CNPs as an example, the 3D RVE model is shown in Figure 6. The displacement load U was applied at vertex C along the Y direction. The displacement constraints were applied in the *X*, *Y*, and *Z* directions at vertex O, and in the *Y* and *Z*, *X* and *Y* directions at vertices A and B, respectively. The deformation of the model was controlled using the reference points A, B, and C, which are kinematically coupled with the RVE surfaces in the loading direction.

**Figure 7:**
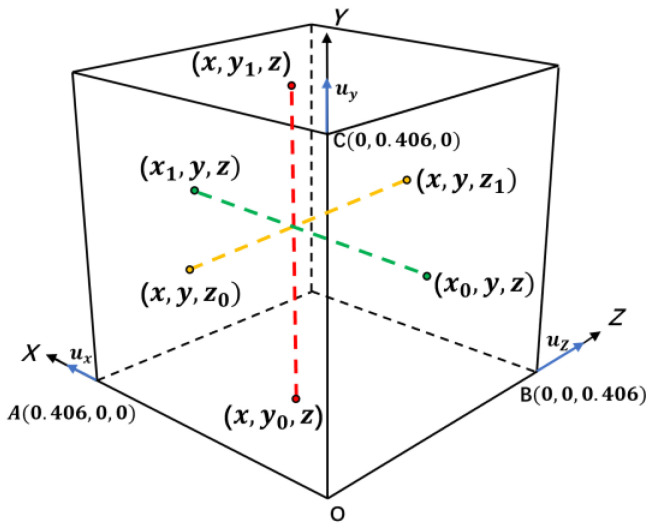
Illustration of the periodic boundary condition.(The dashed lines represent the mutual constraint relationship between two corresponding nodes on two corresponding surfaces.)

To ensure the continuous displacement of the RVE model, periodic boundary conditions must be imposed in the *X*, *Y*, and *Z* directions. As shown in Figure 7, the nodes on the two corresponding boundary surfaces of the model were one−to−one, such as the nodes $\left(x,y,z_{0}\right)$ and $\left(x,y,z_{1}\right)$($z_{1}=z_{0}+0.406$) at the top and bottom of the model. The deformations of the corresponding nodes on the surfaces were always consistent during loading. Hence, the corresponding periodic boundary conditions can be expressed in vector form as

(5) $$\left\{\begin{array}{c} u_{\left(x_{0},y,z\right)}=u_{\left(x_{1},y,z\right)}-u_{x}\\ u_{\left(x,y_{0},z\right)}=u_{\left(x,y_{1},z\right)}-u_{y}\\ u_{\left(x,y,z_{0}\right)}=u_{\left(x,y,z_{1}\right)}-u_{z} \end{array}\right.$$

where $u_{x}$, $u_{y}$, and $u_{z}$ represent the displacement vectors along the *X*, *Y*, and *Z* directions, and they are determined by the loading conditions and the positional relation between the nodes and the loading location.

In the RVE model, the aggregates were treated as rigid bodies since the Young’s modulus of CNPs is much greater than that of the matrix. Due to the hyperelasticity of the silicone rubber under large deformation, the constitutive model of the matrix adopted the Yeoh model, which has been proven to be more suitable for the large deformation of CNPs−filled rubber [32]. For each group, five random RVE models were generated by improved NNA, respectively, to reduce the errors and improve the accuracy of the simulation. Each model was simulated by FE analysis, and the average resistance was calculated.

**Figure 8:**
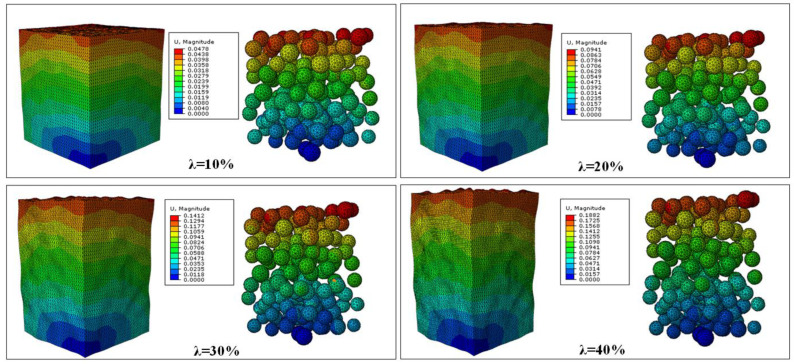
Displacement distribution of the 3D RVE model of EC−300J CNPs−filled silicone elastomer with 30% volume fraction at different uniaxial elongations λ.

Taking the silicone elastomer filled with EC−300J CNPs with 30% volume fraction as an example, the results of the FE analysis of the RVE model are shown in Figure 8. It can be seen that the matrix was gradually stretched along the loading direction with elongation, while it was compressed in the other two directions. Hence, although the position of the aggregates in the matrix changed, the overall junction widths of the aggregates increased along the loading direction and decreased in the other two directions. From the results of the FE analysis, the variation of the junction width can be extracted, by which the unit cell resistances $h_{i}$,$v_{i}$,$d_{i}$, and $w_{i}$ in Figure 5 can be determined using Equation (4). Then, the equivalent resistance of the circuit network of composite can be derived using the admittance matrix method [33]. Based on Kirchhoff’s law, the recurrence formula between the admittance matrix $A_{L+1}$ and $A_{L}$ is as follows:

(6) $$A_{L+1}=\left(A_{L}+D\right){\left(I+Q+HA_{L}\right)}^{-1}M+P$$

**Figure 9:**
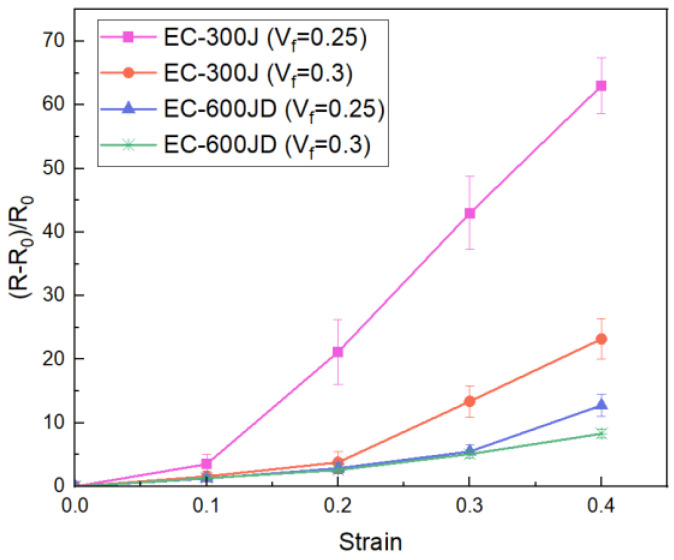
Predicted electromechanical responses of CNPs−filled silicone composite with EC−600JD and EC−300J fillers under uniaxial tensile loading (*V_f_* is the volume fraction).

Here, matrices *D*, *Q*, *H*, and *P* are related to unit resistances $h_{i}$,$v_{i}$,$d_{i}$, and $w_{i}$. It is assumed that the material is uniformly distributed along the thickness direction, so the resistivity of the three−dimensional equivalent circuit network can be simplified as follows:

(7) $$\eta_{3}=\frac{T_{k}\times L\times \left(\bar{\Delta }+d_{m}\right)}{\bar{\Delta }\times N\times A_{L}\left(1,1\right)}$$

where $\bar{\Delta }$ represents the average distance between adjacent aggregates, $d_{m}$ stands for the average diameter of aggregates, $A_{L}(1,1)$ is the first element of the admittance matrix, $T_{K}$ is the thickness of the composite, and *N* and *L* are the numbers of nodes in two directions of the xy plane, respectively. The predicted electromechanical responses of the CNPs−silicone composites with EC−600JD and EC−300J fillers at different volume fractions are shown in Figure 9. The results show that the relative resistance of the composite changes nonlinearly with the increase of strain, and the change of resistance will slow down with the increase of CNPs.

## 4. Verification of the Method

### 4.1. Materials

To verify the predicted results, the silicone elastomer filled with EC−300J and EC−600JD CNPs were produced, respectively. The matrix was room−temperature vulcanized methyl silicone rubber, which had a viscosity of 10,000 cs and good insulation. Ketjenblack EC−600JD or EC−300J were uniformly mixed into the matrix as fillers according to a certain weight ratio, respectively. In addition, dimethyl silicone oil, ethyl orthosilicate, coupling agent, and other auxiliary chemical reagents were required. 

**Figure 10:**
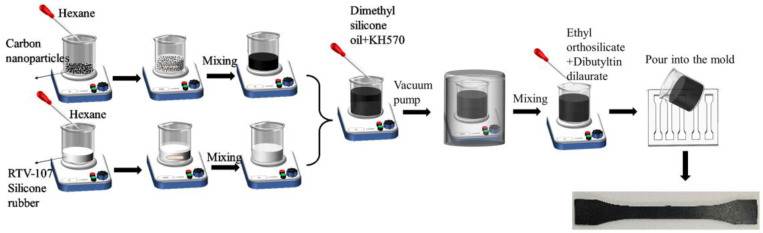
Preparation process of the CNPs−filled silicone rubber.

**Figure 11:**
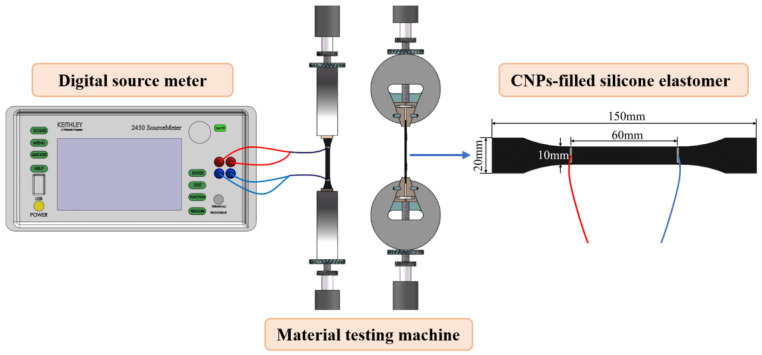
Schematic diagram of the test setup and specimen.

The samples were prepared with the solution mixing method and the process is shown in Figure 10. First, the conductive filler was added to the hexane solvent and stirred. Subsequently, 100 phr of silicone rubber was added to the solvent and thorough stirring was continued. Then, 5 phr dimethyl silicone oil, which is the diluent, and 3 phr silane coupling agents were mixed into the solution. After the solution was fully mixed, it was put into a vacuum pump to remove the internal bubbles. Subsequently, the appropriate amounts of crosslinking agent and catalyst were uniformly added into the solution, and the mixed solution was poured into the mold. After waiting 24 h at room temperature to complete natural vulcanization, then the dumbbell−shaped test samples with a thickness of 2 mm could be obtained. Finally, the silver wires were attached to the samples with conductive adhesive to facilitate resistance measurement. The size of the sample is shown in Figure 11.

### 4.2. Uniaxial Tensile Test

Tensile tests were performed on a material testing machine (Instron−5848) (as shown in Figure 11). The specimen was fixed on a clamp and connected to the digital source meter (Keithley 2450) with conductive wires, while insulation treatment was carried out between the specimen and the clamping head. The electrical resistance of the specimen was measured using the four−probe method, and the gauge length was 60 mm. The displacement was tested by the electronic clip−on extensometer. Displacement loading was supplied on the specimen with a speed of 4 mm/min to ensure the specimens extended under a quasistatic state.

### 4.3. Results and Discussion

**Figure 12:**
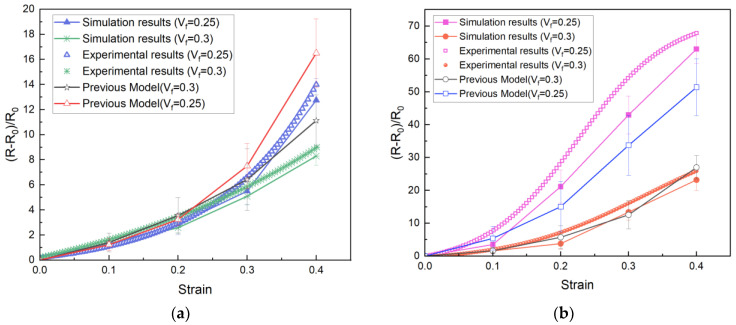
Comparison of the predicted results and the experimental results: (**a**) Silicone elastomer filled with EC−600JD CNPs; (**b**) Silicone elastomer filled with EC−300J CNPs.

The comparison of the simulation results and the experimental results is shown in Figure 12. The calculation results with the 2D RVE model in our previous work are also presented for comparison. It was found that the prediction of the trend of the electromechanical response with the improved method was in good agreement with the experimental results. Compared to the previous model, the simulation results of the 3D RVE model were closer to the experimental data and had less discreteness. The resistances of the elastomers changed nonlinearly with increasing strain, especially under large strain. In comparison, the resistance of the elastomer with EC−300J fillers increased more obviously than that of the EC−600JD fillers. That is because EC−600JD CNPs have a higher specific surface area, smaller particle size, and greater porosity, so the aggregates can form a stronger and more developed branched chain network more easily.

For the same conductive filler, the resistance decreased with the increase of filling amount, but the linearity of the relative−resistance−change−rate–strain curve was improved. The reason is that when increasing the amount of filling of CNPs, the change space of the position of the aggregates during loading is limited, so the variation of junction widths between the aggregates also becomes slow.

Although the simulation results were closer to the experimental data, there was still some error in the values, especially for the silicone elastomer filled with EC−300J CNPs under high strain. This is mainly attributed to the assumption that the aggregates do not change during deformation. The CNP aggregates in the polymer will recombine during loading; that is, some particles can possibly become separated from the aggregate along the tensile direction, and some neighboring aggregates can become close to each other until a new aggregate is formed in the perpendicular directions. The reorganization of the aggregates will lead to a different conductive network, especially for large deformations. In addition, the simplification of the equivalent resistance calculation will also bring some errors.

## 5. Conclusions

In this paper, a multiscale analysis method is proposed to simulate the electromechanical behavior of CNPs−filled polymers, which have been widely used as strain sensors. A 3D RVE model generated by improved NNA was used to simulate the large deformation of the composite under tensile loading. Compared to the previous model, the 3D RVE model contains more details and higher randomness, and is closer to the real composite structure. Then, through FE analysis, the junction widths of the neighboring aggregates in the model were obtained. In addition, the CNPs−filled polymer was simplified as a 3D infinite circuit network composed of numerous random resistors whose tunneling resistance depended on the junction widths of the aggregates. Therefore, by the admittance matrix method, the equivalent resistance of the circuit network was calculated.

Using the proposed method, we predicted the relationship between the resistance and strain of the CNPs−filled polymer with different types of filler under different filling amounts. Meanwhile, the experiment was carried out to verify the simulation results. Due to the assumptions and simplifications of the model, there were some errors between the simulation and experimental results, especially for the lower filling amount and the higher strain. Nevertheless, this multiscale analysis method can not only reveal the strain sensing mechanism from the microscale, but also predict the sensing behavior of CNPs−filled polymer with different material parameters, which is of great significance for the fabrication and improvement of the highly stretchable polymer sensor.

## Figures and Tables

**Table 1:** Material parameters of CNPs.

CNPs	Diameter (nm)	Density (kg/m^3^)	Surface Area (m^2^/g)	DBP Absorption (cm^3^/100 g)
EC−600JD	30	110	1400	510
EC−300J	50	130	800	380

**Table 2:** Material parameters of silicone rubber.

Young’s Modulus (MPa)	Density (kg/m^3^)	Tensile Strength (Mpa)	Elongation at Break (%)
0.85	980	1.6	160

## Data Availability

The data presented in this study are available on request from the corresponding author.
